# 

**Published:** 1904-03-15

**Authors:** 


### CONTENTS.

Amalgam, - - - *By H. P. Martin, D.D.S.* 109
Helps in Practice-Building, *By A. J. Flanagan, D.D.S.* 115
The Evil Effects of Some Dentifrices, *By F. J. Turnbull* 122
Shall the London University Institute a Degree in Dentistry? 127
A Four-Year Course or a Three-Year Course? *By Frank L. Platt, D.D.S.* 135
Porcelain Crowns, - *By F. Ewing Roach, D.D.S.* 138
Identification of Iroquois Fire Victims, *By Geo. W. Cook, D.D.S.* 141
Criticism of the National Association of Dental Faculties 142
Two Cases of Replantation, *By J. H. Gibbs, L.D.S.* 144
EDITORIAL, - - - - - - - - - - 148
ANNOUNCEMENTS, - - - - - - - 150
OBITUARY, - - - - - - - - - - 152
INDENTS, - - - - - - - - - - 153

